# A microcomputer- controlled thermostat

**DOI:** 10.1155/S146392468100059X

**Published:** 1981

**Authors:** Anders Graneli, Olof Johansson, Mats Strandberg, Claes Svensson, Margareta Wedborg

**Affiliations:** 1 Department of Analytical and Marine Chemistry Chalmers University of Technology and University of Göteborg Göteborg S-412 96 Sweden; 2 Astra AB Söderälje S-151 85 Sweden; 3 Supra AB Storgatan 24 Fack Landskrona S-261 Sweden


### A microcomputer- controlled thermostat

**Anders Graneli*, Olof Johansson, Mats Strandberg*, Claes Svensson** and Margareta Wedborg**
*Department of Analytical and Marine Chemistry, Chalmers University of Technology and University of Göteborg, S-412 96 Göteborg, Sweden*

### Introduction

Careful temperature control is essential for a wide variety of chemical measurements. High-precision potentiometry, for instance, requires thermostatting, since the slope of the straight line E vs log $a_i$ is highly temperature dependent (E is the measured emf, log $a_i$ is the logarithm of the activity of species i). The equation describing the relationship between the measured quantity and the activity of species is:

$$E = E_k + RT\ln 10/(nF)\log a_i$$

where $E_k$ includes the normal potential as well as the medium effects, n is the number of electrons transferred in the redox process (or the charge of species i in the case of a membrane electrode), F is Faraday's constant and T is the absolute temperature. Differentiation of this equation with respect to the absolute temperature gives [1]:

$$(dE/dT) =$$

$$(dE_k/dT) + R\ln 10/(nF) \log a_i + RT\ln 10/(nF)(d \log a_i/dT)$$

Separation of the three terms contributing to temperature dependence is difficult in practice, but it is generally considered that the first two terms are dominant and in the order of 1 mV · $K^{-1}$ [2]. Accordingly, the temperature in a potentiometric measurement where a voltmeter with a resolution of 0.01 mV is used should preferably not vary more than ± 0.01 K in order to make full use of the resolution.

Usually thermostats consist of a large oil or water bath which is equipped with a heater and a cooler and controlled by a contact or resistance thermometer. The relatively large volume of water or oil (typically 5-30 l) results in a substantial heat capacity, and as a consequence, such a system is not very sensitive to perturbations. However, what is advantageous with respect to the stability of the system, becomes a disadvantage as soon as change of temperature is required. In such cases, oil or water bath systems are sluggish. The time necessary to achieve stable temperature after a change of temperature of 20°C is often in the order of 24

* *Present address: Astra AB, S-151 85 Södertälje, Sweden*
** *Present address: Supra AB, Storgatan 24, Fack, S-261 Landskrona, Sweden*

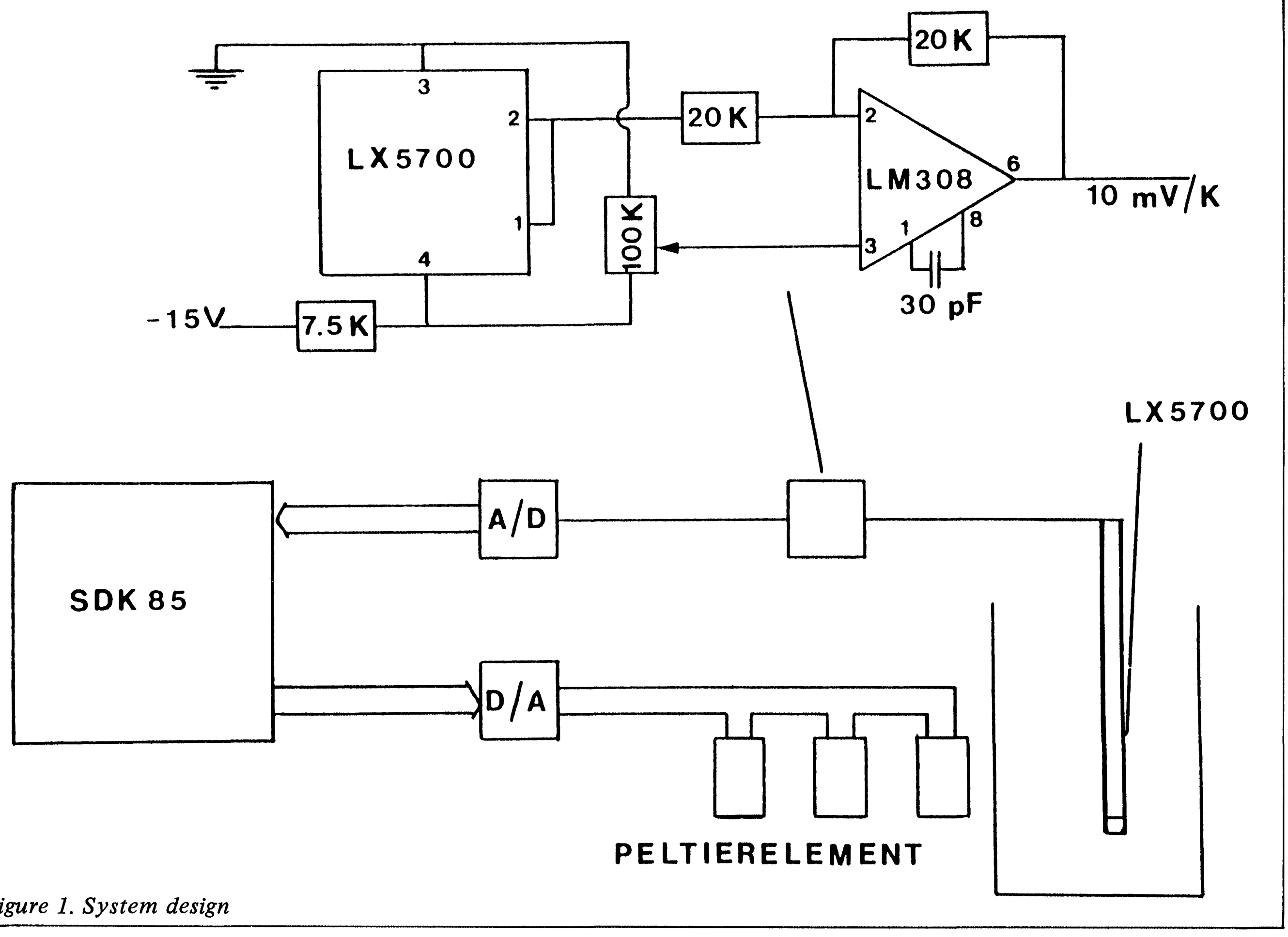

*Figure 1. System design*

hours. In addition, when cooling is required to maintain the temperature close to 0°C, large compressor coolers must be used and the time necessary to attain stable temperature could be a matter of days.

A quite different approach to the thermostatting problem is to use commercially available Peltier elements, which essentially work as electronic heat pumps, moving heat from one side of the element to the other. Such elements can be attached directly to the vessel to be thermostatted, thus making the thermostat bath superfluous. As a result, substantial changes of temperature can be performed very rapidly, in the order of ten minutes for a titration vessel containing 100 ml of aqueous solution. Since requirements for the thermostat system are that changes of temperature between +5°C and +40°C should be performed quickly and easily, this latter approach was chosen for the construction of the system.

### Description of the system hardware

### Thermometer

A temperature sensitive integrated circuit was used (National Semiconductor NS LX5700), designed to give a change of output of 10 mV · $K^{-1}$. The sensor, which has a diameter of ca 5 mm, was mounted inside a plexiglass tube, at the extreme bottom end. A piece of plexiglass was glued on to seal the tube. Near the sensor, the plexiglass was ca 0.5 mm thick. The temperature sensor was connected to a digital panel meter with a resolution of ±0.1 mV (Newport 2000A-2) and calibrated against a certified thermometer.

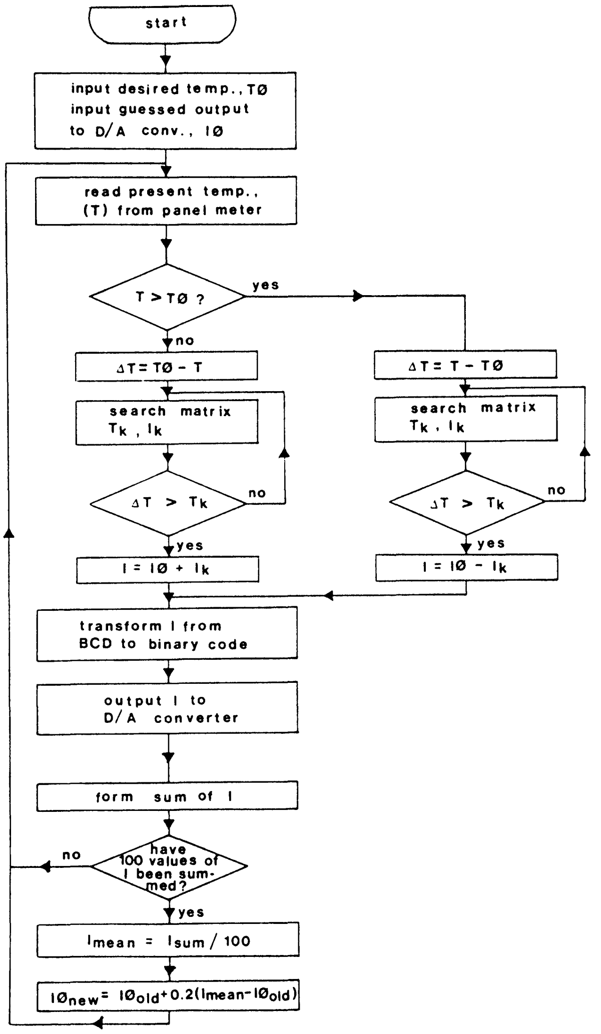

*Figure 2. Flowchart of the program.*

### Temperature control system

An Intel SDK-85 microcomputer kit comprising an 8085A CPU, expanded with 1 kbyte RAM was used. Since the drive capability of the output port of the SDK-85 was too small, the system was also equipped with buffer circuits between the SDK-85 and the D/A-converter.

### Power source

A 12-bit digital-analog current source delivering a maximum of ±10 A at 12 V to the heater-cooler circuits was used.

### Heat source

Three Nortron Peltier-Kühlblocks (PKE 36 A 001) each delivering a cooling effect of 20 W and a heating effect of approximately 40 W were used. Tap water was used for cooling. The difference between heating and cooling effect is caused by the ohmic loss, which adds to the heat transfer effect.

### I/O handling

In the basic version of SDK-85, four eight-bit and two six-bit ports are available. The temperature readings were input to the computer as four parallel BCD-coded digits via two of the eight-bit ports. Output to the D/A-converter was given as a binary twelve-bit pattern via the remaining two eight-bit ports:

| | | |
|---|---|---|
| 1111 1111 1111 (binary), | 4095 (decimal) | - maximum heating current |
| 0000 0000 0000 | , 0 | - maximum cooling current |
| 1000 0000 0000 | , 2048 | - zero current |

### Titration vessel

This was made of metal (gold-plated copper or Teflon covered aluminium) in order to obtain good heat conductivity and designed to contain 160 ml of solution. The system is shown in Figure 1.

### Description of the system software

The control program, about 300 instructions, was written in assembly language. When transferred into machine code, it occupied 600 bytes of memory. The program works according to the following general outline.

The observed temperature, T, is read from the panel meter and a proper output current, I, is calculated and output to the heater-cooler circuits via the D/A-converter. This procedure is repeated ten times per second.

The output current, I, is calculated from:

$$I = I\text{Ø} + \Delta I$$

where IØ is a value for the current corresponding approximately to the desired temperature TØ and ΔI depends on the deviation from the desired temperature, ΔT - T-TØ. Values of ΔT and corresponding values of ΔI are stored in a matrix, which is systematically searched until the first value of ΔT which is smaller than the observed difference is found. The matrix holding ΔT and ΔI is shown in Table 1.

**Table 1. Matrix holding ΔT and ΔI**

| | ΔI (decimal) |
|---|---|
| ΔT>20 (mV) | 4000 |
| 20>ΔT>15 | 2000 |
| 15>ΔT>10 | 1000 |
| 10>ΔT> 5 | 500 |
| 5>ΔT> 2 | 200 |
| 2>ΔT> 1 | 100 |
| 1>ΔT> 0.7 | 70 |
| 0.7>ΔT> 0.3 | 30 |
| 0.3>ΔT> 0.0 | 5 |
| ΔT = 0 | 0 |

The desired temperature, TØ , and the corresponding value for the current output, IØ , are given as inputs at the beginning of program execution. However, the best value of IØ is generally not known, since the system is not isolated and heat transfer between the thermostat and the surroundings changes with room and coolant (tap water) temperature. Also, in the application to titration systems, the heat content of the titration vessel is affected by the addition of titrant, which sometimes deviates substantially in temperature from the titration vessel. Consequently, the input value of IØ is, at best, a fairly good guess, and the system must be adaptive. For this purpose, the program calculates new values of IØ , based on the following principle. First the system is allowed five minutes which in most cases is enough to get close (within a few tenths of a degree Kelvin) to the desired temperature. Then a new value of IØ is calculated every 20th second according to the empirical algorithm:

$$I\emptyset(\text{new}) = I\emptyset(\text{old}) + 0.2(I(\text{mean}) - I\emptyset(\text{old}))$$

where I(mean) is the arithmetic mean of the last 100 output current values. The factor 0.2 is a damping factor, preventing large oscillations. If the damping factor is set equal to 1, the system starts to oscillate as much as ±1 K around TØ. As the damping factor is decreased, the response becomes slower. Empirical tests revealed that a value of 0.2 gave a sufficiently fast response and acceptably small oscillations for our purpose. It should be mentioned, however, that the optimum value might be slightly different.

From the above description it is clear that this control system responds to the difference between actual and desired temperature as well as adapts to changes by adjusting the output current. Thus it works in a way similar to a proportional-integral control system.

A flow-chart of the program is shown in Figure 2.

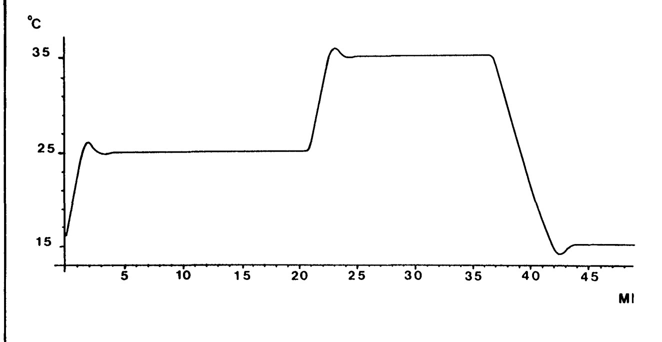

*Figure 3. Temperature response for a sample of 100 ml.*

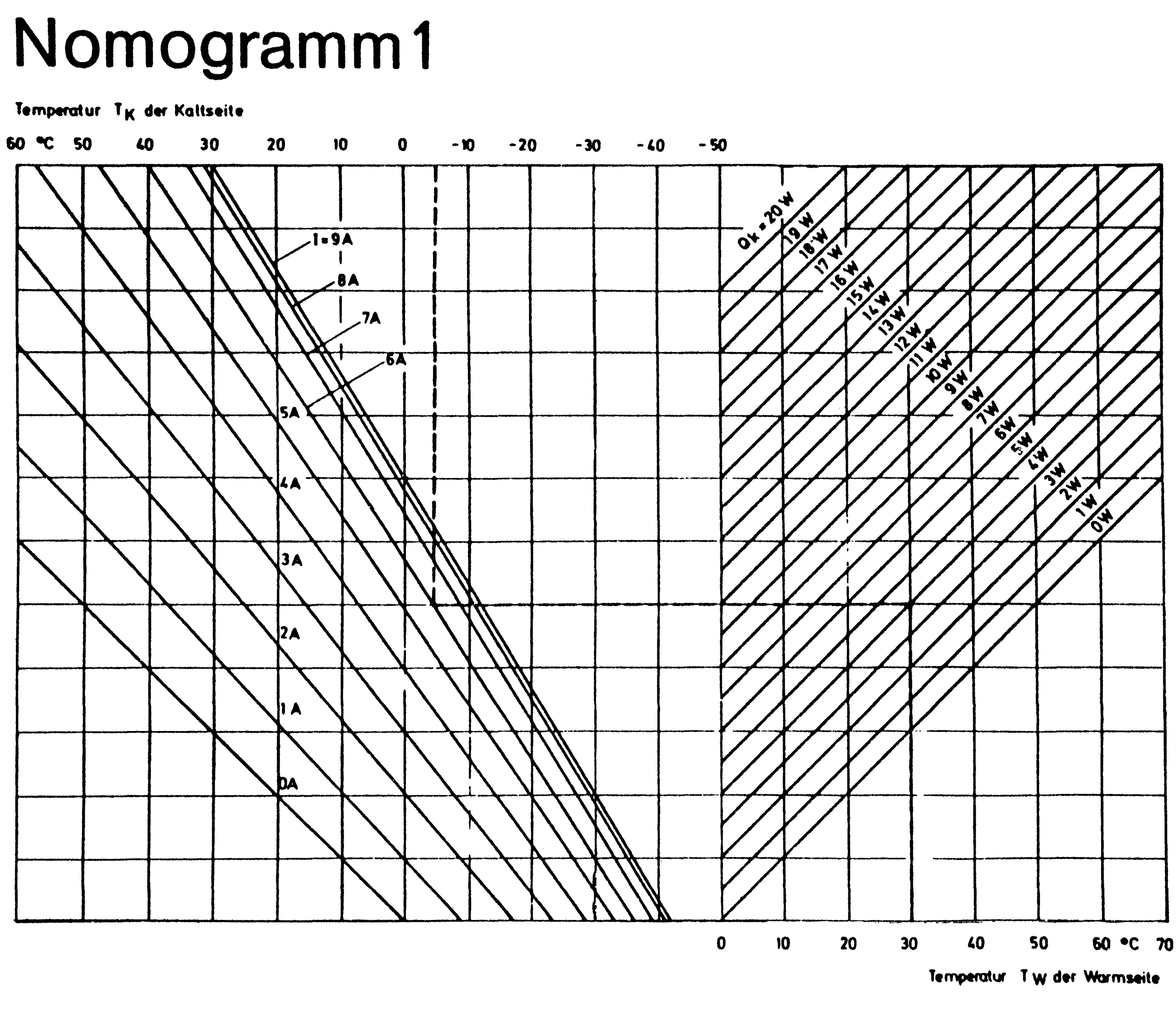

*Figure 4. Nomogram showing the relations between the cold side temperature, the warm side temperature, the current through the Peltier element and the heat-pump effect. When three of these parameters are known, the fourth can be obtained from the nomogram. (Taken from Nortron data sheet).*

### Results and discussion

The system described satisfies to the following requirements:

1. Deviation from desired temperature is ±0.01 K.
2. Rapid attainment of desired temperature. When working at low temperatures at least a factor of 100 faster than conventional thermostats.
3. Temperature programming is easily performed.
4. Does not introduce noise into the measuring system.

The time necessary to reach the desired temperature within ±0.01 K depends on the magnitude of the change of temperature. A change of 10 K requires ten minutes, while a larger change, eg 20 K, can be performed within 15 to 20 minutes. An example is shown in Figure 3.

According to the specification of the Peltier circuits, the maximum temperature difference that can be obtained between the two sides of the circuit is approximately 60 K, ie the temperature of the thermostat can vary within ±60 K from the temperature of the coolant medium. The reason is that at 60 K the normal heat transfer through the Peltier elements due to the temperature difference is equal to the heat transfer produced by the Peltier effect (see Figure 4). Thus the net heat pump effect is zero when the temperature difference between the hot and cold sides is 60 K. To put it more generally, the maximum temperature difference that can be obtained depends on how well isolated the system is. It should be pointed out that the upper limit is set by the fact that the circuits are not designed to resist temperatures higher than +70°C (90°C for short periods). Since this system uses tap water of 15-20°C as coolant, and since it is not well isolated, the temperature range is roughly -30 to +70°C.

Most conventional thermostats use alternating current and generally they are controlled in such a way that all of the heating current is switched on and off. Such a system is a possible source of noise, which might disturb the measuring system. In the case of potentiometric titrations where high impedence electrodes are used, care must be taken to shield the measuring system from the noise generated by the thermostat. With the approach described here, this source of noise is eliminated. The system is driven by direct current, and when the desired temperature is attained, only very small changes of current are needed to maintain the temperature constant.

REFERENCES

[1] Light, T.S. in "Ion-Selective Electrodes" Ed Durst, R.A., 1969, Nat Bur Stand (US), p 354.

[2] Bates, R.G. in "Determination of pH", 1973, Wiley.

### Calendar

**Editor's Note:**
*Organisers of conferences, seminars etc. should send details for inclusion in this calendar as soon as the relevant information is available and not later than three months before the event.*

**Chemistry and analysis of hydrocarbons in the environment, workshop**
November 26-27, Barcelona
*J. Albaiges, Expoquimia, Plaza de Espana, Barcelona-4, Spain*

**In-stream and on-line particle size analysis**

December 2, London

*Dr N.G. Stanley-Wook, University of Bradford, Bradford*

**Capillary columns**
December 3, London
*Miss P.E. Hutchinson, Royal Society of Chemistry, Burlington House, London W1*

**Using microcomputers and microprocessors in the laboratory**
December 4, Chepstow
*Miss P.E. Hutchinson, Royal Society of Chemistry, Burlington House, London W1*

**Computer aided chemistry, specifically related to chromatography and spectroscopy**
December 7-8, London.
*Dr B.P. Chadburn, Perkin-Elmer Ltd, Post Office Lane, Beaconsfield, Bucks*

**Recent advances in analytical chemistry**
December 9-11, London
*Executive Secretary, The Royal Society, 6 Carlton House Terrace, London, SW1Y 5AG*

**Photoacoustic imaging**
December 10, London
*Dr B.C. Beadle, Scientific Conference Services Ltd, 14 Trading Estate Road, London, NW10 7LU*

**Computer aided chemistry, specifically related to chomatography and spectroscopy**
December 10-11, Manchester
*Dr. B.P. Chadburn, Perkin-Elmer Ltd, Post Office Lane, Beaconsfield, Bucks*

**Atomic absorption and emission spectrometry, course**
December 14-18, Loughborough
*Dr J.F. Tyson, Dept of Chemistry, University of Technology, Loughborough Leics, LE11 3TU*

**Moisture measurement seminars**
December 19 & January 13, Luton
*Anachem Ltd, 15 Power Court, Luton, Beds, LU1 3JJ*

**1982**

**Plasma spectrochemistry, 1982 Winter Conference**
January 4-9, Florida
*Conference Chairman, c/o ICP Information Newsletter, Dept of Chemistry, GRC Towers, University of Massachusetts, Amherst, Mass 01003, USA*

**Microprocessor familiarisation course**
January 25-26, Sevenoaks
*Frankie Kingston, Sira Institute Ltd, South Hill, Chislehurst, Kent BR7 5EH*

**The Pittsburgh Conference,**
March 8-12, Atlantic City,
*Pittsburgh Conference, Department J-168, 437 Donald Road, Pittsburgh, PA 15235, USA*

**12th Annual Symposium on the Analytical Chemistry of Pollutants**
April 14-16, Amsterdam.
*Prof Dr. R.W. Frei, Congress Office, 12th Annual Symposium on the Analytical Chemistry of Pollutants, Congress Bureau, Vrije Universiteit, PO Box 7161, 1007 MC Amsterdam, The Netherlands.*

**International Congress on Automation in the Clinical Laboratory**
April 19-22, Barcelona, Spain.
*Dr. R. Galimany, Departmento de Analisis Clinicos, Seccion de Automatizacion, Cuidad Sanitaria 'Principes de Espana', Hospitalet de Llobrogat, Barcelona, Spain.*

**Analytica '82**
April 27-30, Munich
*ECL Exhibition Agencies Ltd, 11 Manchester Square, London W1M 5AB*

